# Changes in Somatostatin-Like Immunoreactivity in the Sympathetic Neurons Projecting to the Prepyloric Area of the Porcine Stomach Induced by Selected Pathological Conditions

**DOI:** 10.1155/2017/9037476

**Published:** 2017-10-02

**Authors:** Katarzyna Palus, Michał Bulc, Jarosław Całka

**Affiliations:** Department of Clinical Physiology, Faculty of Veterinary Medicine, University of Warmia and Mazury in Olsztyn, Olsztyn, Poland

## Abstract

The aim of the present study was to define changes in the expression of somatostatin (SOM) in the sympathetic perikarya innervating the porcine stomach prepyloric area during acetylsalicylic-acid-induced gastritis (ASA) and experimentally induced hyperacidity (HCL) and following partial stomach resection (RES). On day 1, the stomachs were injected with neuronal retrograde tracer Fast Blue (FB). Animals in the ASA group were given acetylsalicylic acid orally for 21 days. On the 22nd day after FB injection, partial stomach resection was performed in RES animals. On day 23, HCL animals were intragastrically given 5 ml/kg of body weight of a 0.25 M aqueous solution of hydrochloric acid. On day 28, all pigs were euthanized. Then, 14-*μ*m thick cryostat sections of the coeliac-superior mesenteric ganglion (CSMG) complexes were processed for routine double-labelling immunofluorescence. All pathological conditions studied resulted in upregulation of SOM-like (SOM-LI) immunoreactivity (from 14.97 ± 1.57% in control group to 33.72 ± 4.39% in the ASA group, to 39.02 ± 3.65% in the RES group, and to 29.63 ± 0.85% in the HCL group). The present studies showed that altered expression of SOM occurs in sympathetic neurons supplying the prepyloric area of the porcine stomach during adaptation to various pathological insults.

## 1. Introduction

Gastrointestinal (GI) tract possesses two sources of innervation: local neurons placed in the gut wall constituting the enteric nervous system (ENS), as well as extrinsic cell bodies originating in sympathetic, parasympathetic, and sensory ganglia [1–3]. Due to numerous active substances secreted by sympathetic neurons, they are involved in the control of many physiological functions in the GI, including local blood flow, motility, hormone release, or HCL secretion [3, 4]. One of these substances is somatostatin (SOM), whose occurrence has been described in rat, guinea pig, swine, and human sympathetic neurons [5–8]. Somatostatin is a regulatory peptide with two bioactive forms, a 14-amino acid peptide (somatostatin-14) and an amino-terminally extended form (somatostatin-28), acting on a wide array of tissue targets to modulate neurotransmission, exocrine and endocrine pancreatic secretions, gut motility, cell proliferation, and angiogenesis [9, 10]. SOM exerts many physiological functions in the central nervous system (CNS), GI tract, thyroid gland, kidney, and immune systems via interaction with one of five G-protein-coupled somatostatin receptor types (sst 1–5) [11, 12]. It is worth emphasizing that the GI tract is a major target of SOM activity [13]. Recent data also suggest that somatostatin is able to inhibit inflammatory processes and exhibits an analgesic effect via inhibition of the sensitization and activation of nociceptors [14]. It is assumed that SOM suppresses intestinal inflammatory responses and participates in bidirectional communication between neurons and mast cells, as well as modulates gut-associated lymphoid tissue (GALT) activities [13]. This hypothesis is supported by data showing enhanced expression of SOM during inflammatory conditions within the GI tract, including inflammatory bowel diseases (IBD), ulcerative colitis, and chemically induced inflammation of the porcine descending colon [15–17]. The vast majority of available data arises from experiments on pigs. Namely, the domestic pig exhibits a high degree of anatomical, histological, and physiological similarity to humans, especially within the gastrointestinal tract and urogenital and cardiovascular systems [18, 19]. Due to this fact, it is regarded as an excellent animal model for the study of GI disorders in human beings.

Over the last decade, extensive morphological and biochemical research concerning pathological processes within the GI tract has been conducted. It is well-known that stress, unhealthy lifestyles, junk food, chemical food additives,* Helicobacter pylori* infection, and abuse of nonsteroidal anti-inflammatory drugs (NSAIDs) increase the risk of stomach diseases, such as erosions, ulceration of gastric mucosa, and/or gastric hyperacidity [20]. Moreover, disorders such as Zollinger-Ellison syndrome, antral G cell hyperplasia, and gastric outlet obstruction via gastrin hypersecretion cause long-term hyperacidity, leading to chronic ulceration of the gastric and duodenal mucosa [21, 22]. Previous studies revealed that the prepyloric area of the stomach is a predictable place of occurrence of pathological changes, such as erosion, ulcers, and inflammatory condition in course of the gastric disorders [23, 24].

In light of previous studies, GI disorders elicit alterations in the neurochemical phenotype of autonomic and ENS neurons, known as neuronal plasticity. Our previous studies revealed that the coeliac-superior mesenteric ganglion (CSMG) complex is the main source of sympathetic innervation of the porcine stomach [25]. Although the majority of previous investigations pertain to the occurrence and changes in somatostatin-like immunoreactivity in structures of ENS [15], its presence in autonomic nervous systems and the precise role in regulation of pathological processes within the GI tract are not fully explained.

The present study was therefore designed to define chemical expression of somatostatin in the sympathetic perikarya innervating the porcine stomach prepyloric area in the physiological state, during acetylsalicylic-acid-induced gastritis and experimentally induced hyperacidity and following partial stomach resection.

## 2. Materials and Methods

### 2.1. Animals and Experimental Procedures

Investigations were performed on 20 sexually immature female pigs of the Large White Polish breed (approximately 8 weeks old, weighing ca. 20 kg) divided into 4 groups: control animals (C group, *n* = 5), acetylsalicylic acid treated (ASA group, *n* = 5), the partial stomach resection group (RES group, *n* = 5), and animals with hydrochloric acid infusion (HCl group, *n* = 5). All animals were kept under standard laboratory conditions with admission to species-specific chow and water ad libitum. All surgical procedures were carried out in accordance with the Local Ethical Committee in Olsztyn (decision number 05/2010). All possible efforts were made to minimize animal suffering.

Animals were treated with azaperone (Stresnil, Janssen Pharmaceutica N.V., Belgium, 4 mg/kg of body weight, i.m.) 15 min before the injection of the main anaesthetic, sodium thiopental (thiopental, Sandoz, Kundl-Rakusko, Austria; 10 mg/kg of body weight, i.v.). Following median laparotomy, the stomachs were exposed and injected with 50 *μ*l (1 *μ*l per 1 injection) of a 5% aqueous solution of the fluorescent retrograde neuronal tracer Fast Blue (FB, EMS-CHEMIE, GmbH, Germany) into the diamond-shaped part (ca. 4 cm × 4 cm) of the stomach anterior prepyloric walls.

Then, ASA pigs were given acetylsalicylic acid (aspirin, BAYER; 100 mg/kg b.w.) orally 1 h before feeding for 21 days (from 7th day after FB injection). On day 22 of the experiment, during laparotomy the partial resection of the previously FB-injected stomach prepyloric areas was performed with an electrosurgical diathermy (ERBE, VIO 300S) in animals of the RES group. On day 23, animals of the HCl group were reintroduced into a state of general anaesthesia (as described above) and intragastrically given 5 ml/kg of body weight of a 0.25 M aqueous solution of hydrochloric acid using a stomach tube. Gastroscopic examinations (using a video-endoscope Olympus GIF 145 with a working length of 1,030 mm and a 9.8 mm diameter) were performed in animals constituting ASA and HCL groups on the first day of the experiment and on the last day of the study. On day 28, all pigs were euthanized by an overdose of anaesthetic and then transcardially perfused with 4% buffered paraformaldehyde (pH 7.4). Following perfusion, the coeliac-superior mesenteric ganglion (CSMG) complexes were collected and postfixed by immersion in the same fixative for 20 minutes, rinsed in phosphate buffer (pH 7.4) for three days, and then stored in a 30% buffered sucrose solution until sectioning. Gastritis in animals of ASA group was confirmed by histopathological examination of fragments of the prepyloric stomach wall collected after perfusion (using routine histopathological methods).

### 2.2. Immunofluorescence Experiments

14-*μ*m thick cryostat sections of the CSMG complexes were analyzed with an Olympus BX 51 fluorescent microscope (Olympus, Poland), equipped with a filter set suitable for observation of FB to localize and count neurons containing the tracer, and were then subjected to routine double-labelling immunofluorescence. Sections were briefly air-dried at room temperature for 45 min and rinsed in 0.1 M phosphate-buffered saline (PBS, pH 7.4; 3 × 10 min), blocked with a mixture containing 10% horse serum and 0.1% bovine serum albumin in 0.1 M PBS, 1% Triton X-100, 0.05% thimerosal, and 0.01% sodium aside for 1 h at room temperature, rinsed in PBS (3 × 10 min) and incubated overnight with a mixture of two primary antisera raised in different species and directed towards somatostatin (rat, cat. number 8330-0009, AbD Serotec, UK, working dilution 1 : 50) and tyrosine hydroxylase (TH) (marker of noradrenergic neurons) (mouse, cat. number MAB 318, Millipore, USA, working dilution 1 : 200) or PGP 9.5 (used here as panneuronal marker) (mouse, cat. number 7863-2004, Biogenesis Ltd., UK, working dilution 1 : 1000). Primary antibodies were visualized by species-specific secondary antibodies conjugated to Alexa Fluor 488 (donkey anti-mouse IgG, cat. number A21202, Invitrogen, USA, working dilution 1 : 1000) and Alexa Fluor 546 (goat anti-rat IgG, cat. number A11081, Invitrogen, USA, working dilution 1 : 1000) for 1 h at room temperature. After staining, the sections were mounted with carbonate-buffered glycerol (pH 8.6) and cover-slipped.

Standard controls included a preabsorption test for the primary antisera with appropriate antigen (S9129, Sigma, St. Louis, MO, USA), an omission test, and a replacement test (replacement of primary antisera with the corresponding nonimmune sera). These procedures completely eliminate specific staining.

### 2.3. Counting and Statistics

The labelled perikarya were viewed under an Olympus BX51 fluorescence microscope equipped with epifluorescence and appropriate filter sets and photographed with a digital camera connected to a PC and processed with Olympus Cell F image-analysis software (Olympus, Tokyo, Japan). To determine the percentage of the particular neuronal subpopulations, at least 300 FB-positive neurons were counted in the CSMG complexes in each of the studied animals, in sections separated by at least 100 *μ*m (minimum 60 sections per animals). Only neurons with a clearly visible nucleus were scored. Data pooled from all animals groups were statistically analyzed using Statistica 10 software (StatSoft Inc., Tulsa, OK, USA) and were expressed as a mean ± standard error of mean (SEM). Significant differences were evaluated using Student's *t*-test for independent samples (^*∗*^*P* < 0.05 and ^*∗∗*^*P* < 0.001). Additionally, the percentage of SOM-LI neurons in the population of CSMG neurons was analyzed. To evaluate the percentage of SOM-LI neurons in the whole population of the CSMG neurons, at least 1000 PGP-9.5-labelled cell bodies in each animal were examined.

## 3. Results

### 3.1. Immunofluorescence

**Figure 1:**
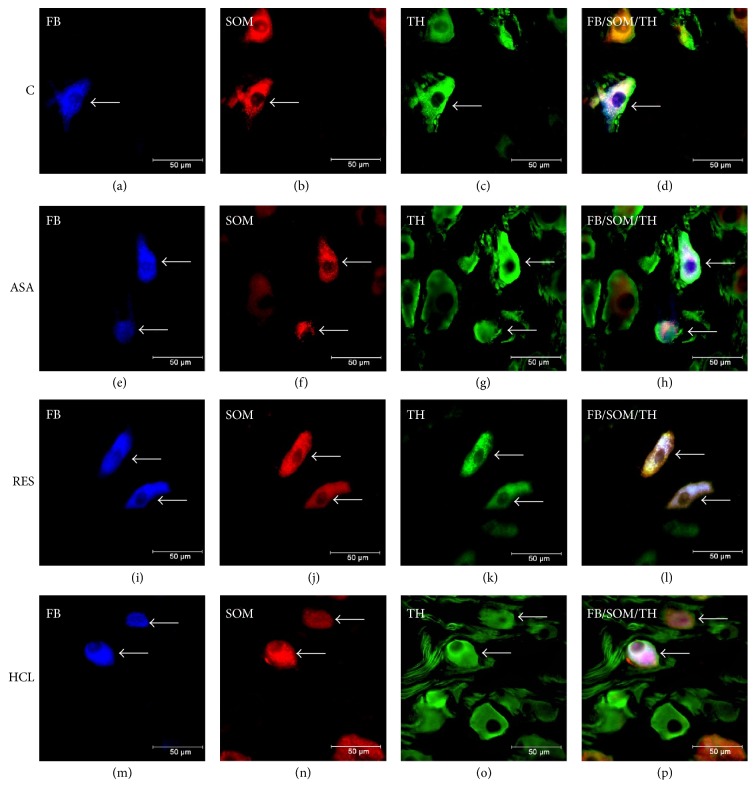
*Representative images of the CSMG neurons projecting to the prepyloric region of the porcine stomach*. Fluorescent micrographs showing FB-labelled neurons (a, e, i, m) displaying immunoreactivity to somatostatin (SOM) and tyrosine hydroxylase (TH) in animals of control (b, c), ASA (f, g), RES (j, k), and HCL (n, o) groups (neurons are indicated with arrows). Photographs (d, h, l, and p) have been created by digital superimposition of three colour channels.

In the control animals, 14.97 ± 1.57% of FB-positive neurons revealed immunoreactivity to SOM (Table 1, Figures 1(a), 1(b), and 1(d)). The results indicate that SOM-LI neurons innervating the prepyloric area of the porcine stomach were distributed exclusively within the area of coeliac poles of the CSMG complex. FB+/SOM+ neurons were randomly distributed in the ganglia and rarely occurred as two cell bodies in the same visual field. The neurons were oval or round, rarely had multipolar shape, and measured 20–40 *μ*m in diameter. All of these neurons were immunoreactive for TH (Table 1, Figures 1(c) and 1(d)). Only single varicose SOM-LI nerve fibres were localized near the FB-positive cells.

None of the studied pathological conditions had an influence on the number of FB-positive neurons supplying the prepyloric area of the stomach but they resulted in upregulation of SOM-LI immunoreactivity in the studied neurons. After ASA-treatment, the number of FB-positive neurons immunoreactive to SOM rapidly increased (to 33.72 ± 4.39%, ^*∗∗*^*P* < 0.001) (Table 1, Figures 1(e), 1(f), and 1(h)). Furthermore, a partial stomach resection resulted in the most remarkable increase in SOM expression in the FB-positive neurons (to 39.02 ± 3.65%, ^*∗∗*^*P* < 0.001) (Table 1, Figures 1(i), 1(j), and 1(l)). Experimentally induced hyperacidity also upregulated expression of SOM in the studied neurons (to 29.63 ± 0.85  ^*∗*^*P* < 0.05) (Table 1, Figures 1(m), 1(n), and 1(p)). In all experimental groups, FB+/SOM+ cell bodies more often formed clusters of 2-3 cells in the same microscopic field. Moreover, all SOM-IR neurons showed TH-immunoreactivity (Table 1, Figures 1(g), 1(k), and 1(o)). The density of SOM-LI nerve fibres did not change significantly compared to control animals.

**Figure 2:**
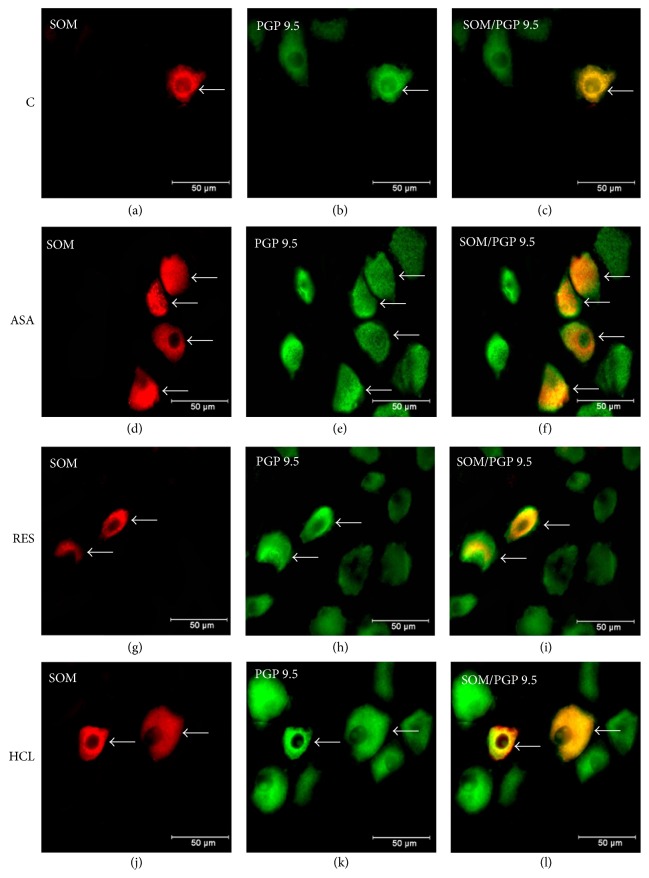
*Representative images of the SOM-LI perikarya in the population of the CSMG neurons*. Fluorescent micrographs showing SOM-LI neurons in the population of the CSMG neurons in animals of control (a), ASA (d), RES (g), and HCL (j) groups (neurons are indicated with arrows). Photographs (c, f, i, and l) have been created by digital superimposition of three colour channels.

Furthermore, in physiological conditions SOM-LI cell bodies represented 12.2 ± 0.72% of the CSMG neurons (Figures 2(a) and 2(c)). The changes observed in experimental groups were not statistically significant (compared to the values observed in control group). In ASA group, we observed 15.8 ± 1.4% of SOM-LI neurons (Figures 2(d) and 2(f)), whereas in RES and HCL groups SOM expression was detected in 14.8 ± 0.7% (Figures 2(g) and 2(i)) and 15.5 ± 0.8% (Figures 2(j) and 2(l)) of the CSMG neurons, respectively.

### 3.2. Gastroscopic and Histopathological Examinations

**Figure 3:**
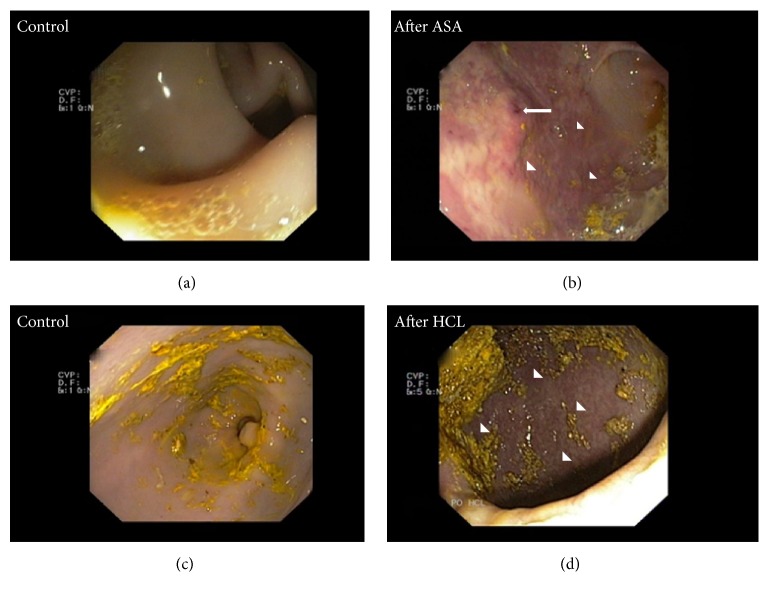
*Gastroscopic examination*. The gastric mucosa without lesions before ASA-treatment (a) and before HCL infusion (c). Hyperaemia (arrowheads) and superficial erosion (arrow) caused by ASA-treatment (b) and hyperacidity (d).

During the gastroscopic examination conducted at the beginning of the study there were no pathological changes in the gastric mucosa in animals of ASA and HCL groups (Figures 3(a) and 3(c)). Long-term administration of ASA triggered hyperaemia and numerous erosions and ulcerations in the mucosal surfaces of the stomach and duodenum (Figure 3(b)). Experimentally induced hyperacidity also revealed pathological changes such as erosions, oedema, hyperaemia, and small ulcers (Figure 3(d)).

**Figure 4:**
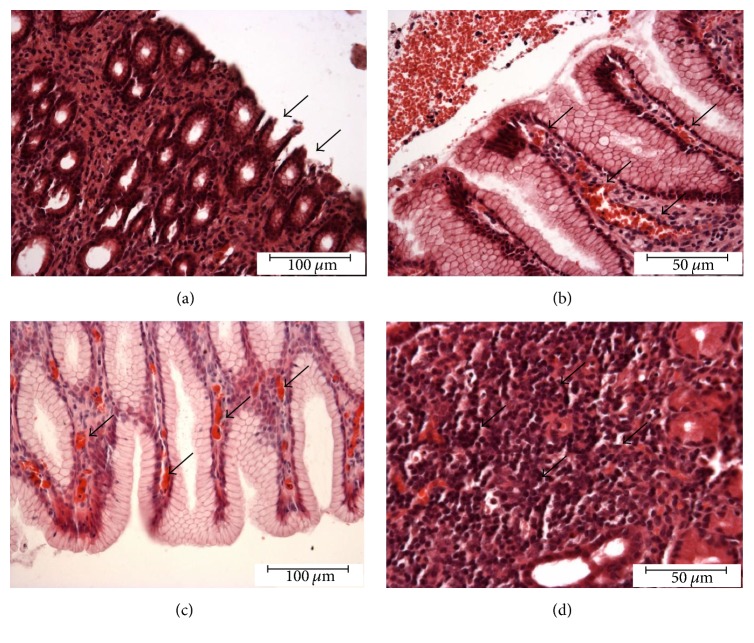
*Histopathological examination*. Histopathological changes in the gastric mucosa induced by long-term ASA-supplementation: superficial erosion (arrows) (a), hyperaemia (arrows) (b, c), and proliferation of lymphatic cells (arrows) (d).

Histopathological examination performed on the wall of gastric prepyloric area collected from animals of the ASA group confirmed gastritis caused by ASA-treatment. Microscopic changes such as superficial erosions, hyperaemia, infiltration of eosinophils, and proliferation of lymphocyte in the gastric mucosa were also observed (Figures 4(a), 4(b), 4(c), and 4(d)).

## 4. Discussion

This is the first report demonstrating the distribution of SOM-LI immunoreactivity in the sympathetic neurons supplying the prepyloric area of the porcine stomach. These results are consistent with the data describing the occurrence of SOM in sympathetic neurons innervating the guinea pig and swine pylorus [8, 26], as well as guinea pig intestine [27], and provide further evidence establishing the important role of SOM in sympathetic innervation of the gastrointestinal tract. However, the number of neurons displaying immunoreactivity to SOM clearly depends on both the animal species and the region of the GI tract studied.

In the view of previous studies, autonomic nervous system exhibits a high degree of plasticity expressed as adaptive changes in the structure and/or function of nerve cells, as well as alterations in the neurochemical phenotype in response to changing environmental conditions [28, 29]. In response to pathological conditions (inflammatory processes, trauma, or toxins), sympathetic neurons particularly increase the expression of biologically active substances which may play a neuroprotective role and promote the regeneration of damaged neurons [29, 30]. This is well in line with the results of this study, where enhanced expression of SOM in the FB-positive CSMG neurons was observed in all experimental groups. This may suggest that SOM is an important neuroprotective factor, which plays a role in survival and regeneration of damaged neurons during inflammation or mechanical injury. The alterations in SOM expression observed in this experiment may be due to changes at the axonal transport stage (inhibition of transport) as well as a result of increased synthesis at various stages of this process (transcription, translation, or changes in the activity of enzymes involved in the synthesis). However, the exact mechanism of these changes has not been fully explained and requires further investigation.

An increase in the population of SOM-LI neurons in animals of the ASA group correlates well with the fact that somatostatin exerts antinociceptive/analgesic and anti-inflammatory effects during inflammatory processes [14, 31], including GI inflammatory pathologies [13]. Recent evidence demonstrates significant changes in SOM expression in idiopathic inflammatory bowel diseases [16], Crohn's disease, ulcerative colitis [17], chemically induces colitis [29], proliferative enteropathy, and chemically driven inflammation of porcine descending colon [15]. SOM modulates inflammatory response by influencing GALT activity, leading to inhibition of cytokine synthesis and release by lymphocytes, lymphoid cell proliferation, and monocyte activity [13]. Using a murine model of colitis, Pintér et al. [32] demonstrated the beneficial effects of SOM and its analogue octreotide on the structure of the mucosa and reduction of inflammatory reaction. Interestingly, somatostatin exhibits the neuronal inflammatory-inhibitory effects via SSTR4 receptor [33]. Long-term administration of ASA applied in this experiment resulted in chronic inflammation of the gastric mucosa, confirmed by using gastroscopy and histopathological studies. It may explain the increase in SOM immunoreactivity in the CSMG neurons constituting the ASA group. Therefore, further research is urgently needed to elucidate the exact mechanism of SOM in neuronal response during ASA-induced gastritis in pigs.

On the other hand, neuronal transection following partial stomach resection also triggered significant changes in SOM-LI immunoreactivity, which is in agreement with previous findings on the influence of axotomy on an increase in SOM expression [31]. In contrast, other authors reported decreased expression of SOM after neuronal injury [34]. Different changes in expression of SOM (increase and decrease) in sympathetic neurons after axotomy may be the result of different research methodology applied, as well as target-dependent neurons responses. However, despite the fact that the precise role of SOM is not fully recognized, it is assumed that its antianalgesic effect is involved. Indeed, somatostatin is known as a strong antinociceptive factor [14, 35]. SOM downregulates nociception by acting prejunctionally on the sensory-efferent nerve terminal and inhibits the release of neurotransmitters involved in pain transmission [32]. Furthermore, after axotomy, sympathetic neurons are deprived of target-derived nerve growth factor (NGF) and are exposed to the cytokine leukaemia inhibitory factor (LIF), both within the ganglion and at the site of the injury, which promotes the expression of vasoactive intestinal peptide (VIP) and galanin (GAL) [36, 37]. These same factors may be responsible for upregulation of SOM expression in the CSMG neurons after injury. It is also important to mention that nerve injury (axotomy, neuronal transection) led to upregulation of the synthesis and release of some bioactive substance conductive to the regeneration and survival of damaged neurons [30, 38]. Previous mentioned studies supported by the results of this experiment suggest the neuroprotective role of SOM in neuronal response to peripheral injury of sympathetic neurons in pigs.

It has been shown that SOM suppresses the synthesis and release of HCL in the stomach. These effects may be achieved by the direct effect of SOM produced by antral D-cells as well as by the neuronal form of SOM [13]. Notably, the presence of this neuropeptide has been described in the population of ENS neurons [15]. It is well-known that sympathetic postganglionic neurons that supply the gastrointestinal tract exhibit their action via cooperation with ENS [39, 40]. The considerable increase in SOM expression in the CSMG neurons supplying the prepyloric area of the porcine stomach during experimentally induced gastric hyperacidity may arise from the augmentation of SOM synthesis in the cell bodies as an adaptive process. Additionally, based on literature data we may speculate that the density of somatostatinergic fibres in the prepyloric wall was also increased. Previous studies in mice, concerning intestinal inflammation due to schistosomiasis, revealed an increased proportion of somatostatinergic spinal neurons projecting to the ileum [41, 42] and an increased density of somatostatinergic fibres in the ileal wall [43].

On the other hand, alterations in SOM expression observed in this study may be triggered by inflammatory changes in the gastric mucosa, which confirmed the results of gastroscopy examination. Namely, ulcers and erosions resulting from gastric hyperacidity may lead to activation of the pain and inflammatory response. This is well in line with previously described anti-inflammatory and antinociceptive effects of SOM. However, the question of the precise mechanism of action of SOM released from sympathetic neurons supplying the prepyloric area of the stomach following hyperacidity is still poorly understood and remains to be elucidated.

One could notice the discrepancy between ratio of increase of the number of SOM-LI neurons in proportion to the FB-labelled or total PGP 9.5-LI neurons in control and pathological groups. Comparably higher proportional increase of the number of the SOM-LI neurons in population of the FB-labelled cells in comparison to the lower proportional increase of the SOM-LI neurons to the total number of PGP 9.5-LI cells results from the fact that increased numbers of SOM-LI cells in the experimental groups are attributed to innumerous population of the FB-labelled cells comparing to numerous group of the PGP 9.5-LI neurons. Acquired data seem to confirm selective response of the stomach projecting neurons (FB-positive) to the local (gastric) inflammatory state and not to the systemic inflammatory conditions, because other CSMG neurons did not respond with overexpression of SOM.

In conclusion, the present study showed that altered expression of SOM occurs in sympathetic neurons supplying the prepyloric area of the porcine stomach during adaptation to various pathological insults (axotomy, inflammatory condition, and hyperacidity). The enhancement in SOM-LI immunoreactivity in all experimental groups supports the existing knowledge of its anti-inflammatory and antianalgesic effects, indicating a neuroprotective role of SOM in different traumatic and inflammatory processes within the GI tract. A very significant increase in SOM immunoreactivity in experimentally induced pathological conditions of the stomach may suggest their SOM-specific character. In the future, somatostatin and its analogues may be used in the restoration of the physiological function of the stomach after bariatric surgery, as well as adjunctive therapy in hyperacidity or inflammation of the gastric mucosa. Up to now, SOM or its analogues are useful medications in treatment of diarrhoea [44]. Therefore, further clinical trials are urgently needed to explore the precise role and usefulness of SOM in the experimentally induced disorders presented in this study.

## Figures and Tables

**Table 1:** The percentages of different populations among FB+ neurons in the CSMG of the control, ASA, RES, and HCL pigs. Data expressed as mean ± SEM. Asterisks mark statistically significant differences at ^*∗*^*P* < 0.05 and ^*∗∗*^*P* < 0.001.

	FB+/TH+/SOM+	FB+/TH+/SOM−	FB+/TH−/SOM+	FB+/TH−/ SOM−
Control	14.97 ± 1.57	79.88 ± 0.7	0	5.15 ± 2.54
ASA	33.72 ± 4.39^*∗∗*^	52.06 ± 2.6^*∗∗*^	0	14.22 ± 1.03^*∗∗*^
RES	39.02 ± 3.65^*∗∗*^	45.54 ± 4.1^*∗∗*^	0	15.44 ± 0.89^*∗∗*^
HCL	29.63 ± 0.85^*∗*^	56.37 ± 1.9^*∗*^	0	14.00 ± 2.87^*∗∗*^
